# High incidence of other primary malignancies in patients with synchronous multiple gastric cancers “a multi-center retrospective cohort study”

**DOI:** 10.18632/oncotarget.25027

**Published:** 2018-04-17

**Authors:** Daisuke Takeuchi, Naohiko Koide, Akira Suzuki, Fumiaki Shimizu, Yoshinori Koyama, Takehito Ehara, Yuta Yamamoto, Makoto Koyama, Satoshi Nakamura, Masato Kitazawa, Yusuke Miyagawa, Shinichi Miyagawa

**Affiliations:** ^1^ Department of Surgery, Shinshu University School of Medicine, Asahi, Matsumoto 390-8621, Japan; ^2^ Department of Surgery, Nagano Prefectural Kiso Hospital, Asahi, Matsumoto 390-8621, Japan; ^3^ Department of Surgery, Shinshu Ueda Medical Center, Asahi, Matsumoto 390-8621, Japan

**Keywords:** multiple gastric cancers, multiple primary cancers, other primary malignancy, gastrectomy, gastric cancer

## Abstract

This study evaluated the relationship between synchronous multiple gastric cancer and other primary malignancies. During 2002–2013, 1094 consecutive surgically treated gastric cancer patients were enrolled. Preoperatively, we performed total colonoscopy and whole-body computed tomography. When malignancies in other organs were suspected, detailed organ-specific examinations were performed. Synchronous multiple gastric cancer occurred in 102 patients (9.3%)which was frequently observed in patients with preoperative other primary malignancies (*p* < 0.001). Preoperative other primary malignancy was an independent risk factor for synchronous multiple gastric cancer (*p* = 0.001; hazard ratio: 2.145, 95% confidence interval: 1.354–3.399) and an independent prognostic factor of overall survival in patients undergoing gastrectomy with curative intent (*p* = 0.021; hazard ratio: 1.481, 95% confidence interval: 1.060–2.070). Thus, patients with preoperative other primary malignancies have a high risk of synchronous multiple gastric cancer. Careful preoperative examination is recommended to improve survival.

## INTRODUCTION

The detection of multiple lesions of gastric cancer (GC) has been increasing along with advances in endoscopic and pathologic examinations. Synchronous multiple GCs (SMGC) have been reported to account for 5–15% of all GC cases [1]. However, accessory lesions are generally not considered as a prognostic factor in SMGC patients [2, 3].

GC patients sometimes present other primary malignancies (OPMs), particularly in the colon [4]. Recent studies have demonstrated an association between SMGC and OPMs, and found that this association was frequently observed in colorectal cancer patients [5, 6]. However, the exact relationship between SMGC and OPMs remains unclear, and it would be of clinical benefit to clarify what types of OPMs occur in SMGC patients.

With this in mind, we performed the present multi-center retrospective cohort study to clarify the relationship between SMGC and OPMs.

## RESULTS

### Patients

**Figure 1:**
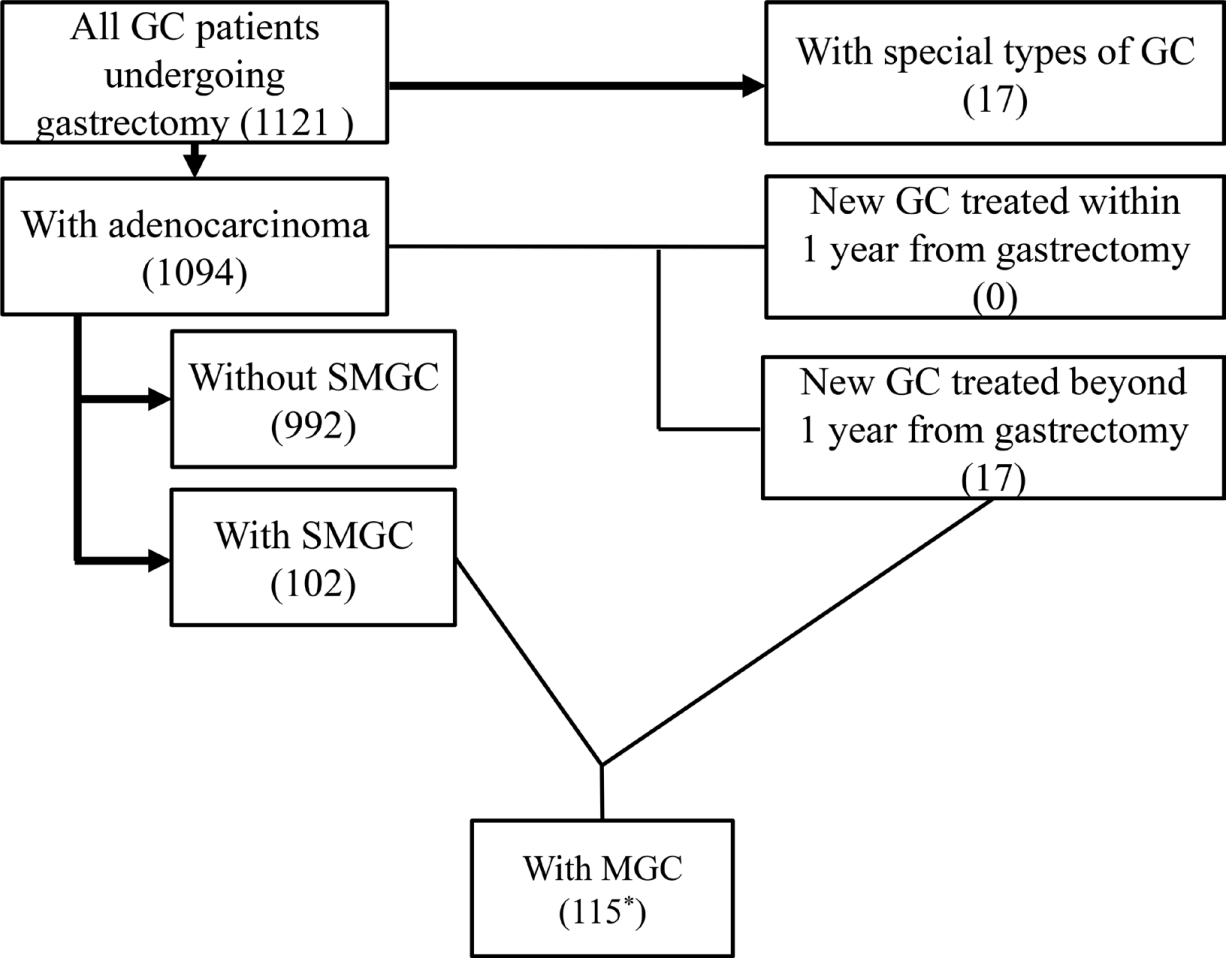
Characteristics of the enrolled study patients A total of 1121 consecutive patients with gastric cancer (GC) were treated surgically between 2002 and 2013. After excluding 17 patients with special types of histology, 1094 patients with gastric adenocarcinoma were enrolled. Seventeen patients had metachronous GC. Including these metachronous GCs, 115 patients had multiple GCs (MGC). ^*^Four patients had both synchronous MGC (SMGC) and metachronous MGC.

The characteristics of the study population are summarized in Figure 1. A total of 1121 consecutive patients with GC were treated surgically between 2002 and 2013 in Shinshu University Hospital, Shinshu Ueda Medical Center, and Nagano Prefectural Kiso Hospital. Excluding 17 patients with special types of histology, such as neuroendocrine carcinoma and squamous cell carcinoma, 1094 patients with gastric adenocarcinoma were enrolled in the present study. Of the 1094 patients with GC, 102 patients (9.3%) had SMGC, including 80 patients with 2 GCs, 18 patients with 3 GCs, and 1 patient each with 4, 5, 6, and 7 GCs. Fifty-five patients underwent gastrectomy after non-curative resection by endoscopic submucosal dissection (ESD), and 34 patients had GC in the remnant stomach. There was no patient with a new GC detected within 1 year after gastrectomy. Seventeen patients had metachronous GC, including 3 patients treated with endoscopic submucosal dissection and 14 patients treated with gastrectomy. Including these metachronous GCs, 115 patients had multiple GC (MGC); 4 patients had both SMGC and metachronous MGC. The mean follow up period was 41.8 months.

### Clinicopathological features of MGC

The clinicopathological features of the patients with or without SMGC are shown in Table 1. SMGC was significantly more frequently observed in elderly patients (*p* = 0.002) and in patients with current smoking (*p* = 0.032), differentiated adenocarcinoma (*p* < 0.001), early cancer (*p* = 0.001), negative node metastasis (*p* = 0.035), negative distant metastasis (*p* = 0.015), and preoperative OPMs (*p* < 0.001) compared to in their counterparts.

**Table 1:** Characteristics of patients with or without synchronous multiple gastric cancer

Variable	With SMGC patients (*n* = 102)	Without SMGC patients (*n* = 992)	*p*-value
Age (years old: mean ± SD)	73.2 ± 10.3	69.7 ± 11.1	0.002
Sex			0.127
male	77 (75.5%)	676 (68.1%)	
female	25 (24.5%)	316 (31.9%)	
Alcohol consumption (every day)			0.275
with	35 (34.3%)	289 (29.1%)	
without	67 (65.7%)	703 (70.9%)	
Current smoking			0.032
with	35 (34.3%)	244 (24.6%)	
without	67 (65.7%)	748 (75.4%)	
Preoperative OPM			<0.001
with	33 (32.4%)	165 (16.6%)	
without	69 (67.6%)	827 (83.4%)	
Tumor location			0.544
upper-third	27 (26.5%)	240 (24.2%)	
middle-third	35 (34.3%)	396 (39.9%)	
lower-third	40 (39.2%)	356 (35.9%)	
Tumor size (mm: mean ± SD)	40.9 ± 26.1	48.6 ± 33.4	0.057
Histologic type			<0.001
differentiated (tub/pap)	77 (75.5%)	551 (55.5%)	
undifferentiated (por/sig/muc)	25 (24.5%)	441 (44.5%)	
Depth of invasion			0.001
pT1	67 (65.7%)	474 (47.8%)	
pT2 or more	35 (34.3%)	518 (52.2%)	
Node metastasis			0.035
pN0	71 (69.6%)	584 (58.9%)	
pN1or more	31 (30.4%)	408 (41.1%)	
TNM Stage			<0.001
I	74 (72.5%)	528 (53.2%)	
II	17 (16.7%)	164 (16.5%)	
III	9 (8.8%)	203 (20.5%)	
IV	2 (2.0%)	97 (9.8%)	
Chemotherapy for gastric cancer			
with	26 (25.5%)	347 (35.0%)	0.054
without	76 (74.5%)	645 (65.0%)	
BMI (kg/m^2^: mean ± SD)	22.6 ± 4.2	22.2 ± 3.3	0.618
Diabetes melites			0.465
with	17 (16.7%)	139 (14.0%)	
without	85 (8.3%)	853 (86.0%)	

SMGC, synchronous multiple gastric cancer; SD, standerd deviation; OPM, other primary malignancy; tub, tubular adenocarcinoma; pap, papillary adenocarcinoma; por, poorly differentiated adenocarcinoma; sig, signet ring cell carcinoma; muc, mucinous carcinoma; BMI, body mass index.

The clinicopathological features of the patients with or without MGC are shown in Table 2. Similar to SMGC, MGC was significantly more frequently observed in elderly patients (*p* = 0.001), men (*p* = 0.036), and in patients with current smoking (*p* = 0.0499), differentiated adenocarcinoma (*p* < 0.001), early cancer (*p* = 0.028), negative node metastasis (*p* = 0.025), and OPMs, including both synchronous and metachronous OPMs (*p* < 0.001), than in patients without these factors.

**Table 2:** Characteristics of patients with solitary and multiple gastric cancer

Variable	With MGC patients (*n* = 115)	Without MGC patients (*n* = 979)	*p*-value
Age (years old: mean ± SD)	73.3 ± 10.2	69.6 ± 11.1	0.001
Sex			0.036
male	89 (77.4%)	664 (67.8%)	
female	26 (22.6%)	315 (32.2%)	
Alcohol consumption (every day)			0.675
with	36 (31.3%)	288 (29.4%)	
without	79 (68.7%)	691 (70.6%)	
Current smoking			0.0499
with	38 (33.0%)	241 (24.6%)	
without	77 (67.0%)	738 (75.4%)	
OPM^*^			<0.001
with	43 (37.4%)	192 (19.6%)	
without	72 (62.6%)	787 (80.4%)	
Tumor location			0.058
upper-third	38 (33.0%)	229 (23.4%)	
middle-third	37 (32.2%)	394 (40.2%)	
lower-third	40 (34.8%)	356 (36.4%)	
Tumor size (mm: mean ± SD)	43.8 ± 29.0	48.33 ± 33.3	0.161
Histologic type			<0.001
differentiated (tub/pap)	84 (73.0%)	544 (55.6%)	
undifferentiated (por/sig/muc)	31 (27.0%)	435 (44.4%)	
Depth of invasion			0.028
pT1	47 (40.9%)	506 (51.7%)	
pT2 or more	68 (59.1%)	473 (48.3%)	
Node metastasis			0.025
pN0	80 (69.6%)	575 (58.7%)	
pN1or more	35 (30.4%)	404 (41.3%)	
TNM Stage			0.001
I	81 (70.4%)	521 (53.2%)	
II	19 (16.5%)	162 (16.5%)	
III	12 (10.4%)	200 (20.4%)	
IV	3 (2.6%)	96 (9.8%)	
Chemotherapy for gastric cancer			0.056
with	30 (26.1%)	343 (35.0%)	
without	85 (73.9%)	636 (65.0%)	
BMI (kg/m^2^: mean ± SD)	22.3 ± 4.2	22.2 ± 3.3	0.835
Diabetes melites			0.463
with	19 (16.5%)	137 (14.0%)	
without	96 (83.5%)	842 (86.0%)	

MGC, multiple gastric cancer; SD, standerd deviation; OPM, other primary malignancy; tub, tubular adenocarcinoma; pap, papillary adenocarcinoma; por, poorly differentiated adenocarcinoma; sig, signet ring cell carcinoma; muc, mucinous carcinoma; BMI, body mass index.

^*^OPM include pre- and postoperative cancers.

### Occurrence of OPMs during the pre- and postoperative period

The most frequently observed malignancy in the total cohort was colorectal cancer, which was observed in 77 patients, followed by prostate and lung cancers. Malignancies of the thyroid (*p* = 0.031), esophagus (*p* = 0.044), and colorectum (*p* = 0.025) were more frequently observed in patients with SMGC than in those without (Table 3).

**Table 3:** Solitary and synchronous multiple gastric cancer patients with other primary malignancies including pre- and postoperatively diagnosed

OPM (pre- and postoperative)	With SMGC patients (*n* = 102)	Without SMGC patients (*n* = 992)	*p*-value
Brain	1 (1.0%)	0 (0%)	0.093
Head and neck			
Oral	1 (1.0%)	0 (0%)	0.093
Laryngopharynx	0 (0%)	12 (1.2%)	0.617
Thyroid	3 (2.9%)	5 (0.5%)	0.031
Chest			
Lung	5 (4.9%)	23 (2.3%)	0.174
Breast	0 (0%)	13 (1.3%)	0.624
Gastrointestinal tract			
Esophagus	4 (3.9%)	11 (1.1%)	0.043
Colorectum	13 (12.7%)	64 (6.5%)	0.025
Hepatobiliary system			
Liver	1 (1.0%)	13 (1.3%)	1.000
Bile duct	0 (0%)	4 (0.4%)	1.000
Pancreas	0 (0%)	2 (0.2%)	1.000
Urogenital system			
Kidney	2 (2.0%)	4 (0.4%)	0.101
Bladder	2 (2.0%)	14 (1.4%)	0.655
Prostate	5 (4.9%)	25 (2.5%)	0.190
Testis	0 (0%)	2 (0.2%)	1.000
Gynecologic organs			
Uterus	0 (0%)	7 (0.7%)	1.000
Ovary	0 (0%)	2 (0.2%)	1.000
Retroperitoneum	1 (1.0%)	0 (0%)	0.093
Skin	1 (1.0%)	4 (0.4%)	0.388
Hematopoietic system	3 (2.9%)	11 (1.1%)	0.135
Soft tissue	1 (1.0%)	1 (0.1%)	0.178
Unknown origin	1 (1.0%)	0 (0%)	0.093
Total	41 (40.2%)	194 (19.6%)	<0.001

OPM, other primary cancer; SMGC, synchronous multiple gastric cancer.

Both antecedent and synchronous OPMs were more frequently observed in patients with SMGC (*p* = 0.032 and 0.006, respectively; Table 4). During the follow-up period, 9 OPMs were detected in 9 patients (8.8%) with SMGC, whereas 38 OPMs were detected in 37 patients (3.7%) without SMGC (*p* = 0.031; Table 4). There was no case of OPM detected within 1 year after gastrectomy.

**Table 4:** Other primary malignancies in patients with or without synchronous multiple gastric cancer

Variable	With SMGC patients (*n* = 102)	Without SMGC patients (*n* = 992)	*p*-value
Antecedant OPM			0.032
with	16 (15.7%)	90 (9.1%)	
without	86 (84.3%)	902 (90.1%)	
Synchronous OPM			0.006
with	17 (16.7%)	84 (8.5%)	
without	85 (83.3%)	908 (91.5%)	
Subsequent OPM			0.031
with	9 (8.8%)	37 (3.7%)	
without	93 (91.2%)	955 (96.3%)	

SMGC, synchronous multiple gastric cancer; OPM, other primary malignancy.

### Relationships between MGC and OPMs

The presence of a preoperative OPM was found to be an independent risk factor for SMGC (*p* = 0.001; hazard ratio [HR]: 2.145, 95% confidence interval [CI]: 1.353–3.399; Table 5), and the detection of an OPM either preoperatively or postoperatively was an independent risk factor for MGC (*p* < 0.001; HR: 2.146, 95% CI: 1.409–3.268; Table 6). Furthermore, patients with current smoking and with SMGC frequently developed OPMs postoperatively (Table 7), and the presence of SMGC was demonstrated to be an independent risk factor for postoperative OPMs (*p* = 0.028; HR: 2.35, 95% CI: 1.095–5.045; Table 8).

**Table 5:** Multivariate analysis for risk factors of synchronous multiple gastric cancer

Variable	*p*-value	Hazard ratio	95% CI
Age (year)	0.002	1.038	1.013–1.063
Current smoking (with)	0.012	1.816	1.139–2.895
Preoperative OPM (with)	0.001	2.145	1.354–3.399
Histologic type (undifferentiated)	0.043	0.601	0.366–0.985
Depth of invasion (pT2 or more)	0.793	0.92	0.494–1.713
Node metastasis (pN1 or more)	0.065	1.794	0.965–3.335
Stage (I, II, III, IV)	0.004	0.503	0.316–0.802

CI, confidence interval; OPM, other primary malignancy.

**Table 6:** Multivariate analysis for risk factors of multiple gastric cancer

Variable	*p*-value	Hazard ratio	95% CI
Age (year)	<0.001	1.039	1.015–1.063
Sex (male)	0.001	1.314	0.801–2.157
Current smoking (with)	0.062	1.553	0.978–2.465
Pre- and postoperative OPM (with)	<0.001	2.146	1.409–3.268
Histologic type (undifferentiated)	0.106	0.683	0.430–1.085
Depth of invasion (pT2 or more)	0.284	1.373	0.769–2.453
Node metastasis (pN1 or more)	0.193	1.503	0.814–2.777
Stage (I, II, III, IV)	0.004	0.47	0.282–0.783

CI, confidence interval; OPM, other primary malignancy.

**Table 7:** Characteristics of patients with and without postoperative other primary malignancies

Variable	With postoperative OPM (*n* = 46)	Without postoperative OPM (*n* = 1048)	*p*-value
Age (years old: mean ± SD)	69.0 ± 7.9	70.1 ± 11.1	0.524
Sex			0.158
male	36 (78.3%)	717 (68.4%)	
female	10 (21.7%)	331 (31.6%)	
Alcohol consumption (every day)			0.433
with	16 (34.8%)	308 (29.4%)	
without	30 (65.2%)	740 (70.6%)	
Current smoking			0.030
with	18 (39.1%)	261 (24.9%)	
without	28 (60.9%)	787 (75.1%)	
SMGC			0.032
with	9 (19.6%)	93 (8.9%)	
without	37 (80.4%)	955 (91.1%)	
Tumor location			0.705
upper-third	12 (26.1%)	255 (24.3%)	
middle-third	20 (43.5%)	411 (39.2%)	
lower-third	14 (30.4%)	382 (36.5%)	
Tumor size (mm: mean ± SD)	42.9 ± 29.1	48.06 ± 33.0	0.297
Histologic type			0.902
differentiated (tub/pap)	26 (56.5%)	602 (57.4%)	
undifferentiated (por/sig/muc)	20 (43.5%)	446 (42.6%)	
Depth of invasion			0.939
pT1	23 (50.0%)	518 (49.4%)	
pT2 or more	23 (50.0%)	530 (50.6%)	
Node metastasis			0.654
pN0	29 (63.0%)	626 (59.7%)	
pN1or more	17 (37.0%)	422 (40.3%)	
TNM Stage			0.057
I	27 (58.7%)	575 (54.9%)	
II	12 (26.1%)	169 (16.1%)	
III	7 (15.2%)	205 (19.6%)	
IV	0 (0.0%)	99 (9.4%)	
Chemotherapy for gastric cancer			0.242
with	12 (26.1%)	361 (34.4%)	
without	34 (73.9%)	687 (65.6%)	
BMI (kg/m^2^: mean ± SD)	22.0 ± 3.0	22.3 ± 3.4	0.646
Diabetes melites			0.535
with	8 (17.4%)	148 (14.1%)	
without	38 (82.6%)	900 (85.9%)	

SMGC, synchronous multiple gastric cancer; SD, standerd deviation; OPM, other primary malignancy; tub, tubular adenocarcinoma; pap, papillary adenocarcinoma; por, poorly differentiated adenocarcinoma; sig, signet ring cell carcinoma; muc, mucinous carcinoma; BMI, body mass index.

**Table 8:** Multivariate analysis for risk factors of postoperative other primary malignancies

Variable	*p*-value	Hazard ratio	95% CI
Current smoking (with)	0.049	1.851	1.004–3.415
SMGC (with)	0.028	2.35	1.095–5.045

CI, confidence interval; SMGC, synchronous multiple gastric cancer.

### Clinical outcomes after surgery

**Figure 2:**
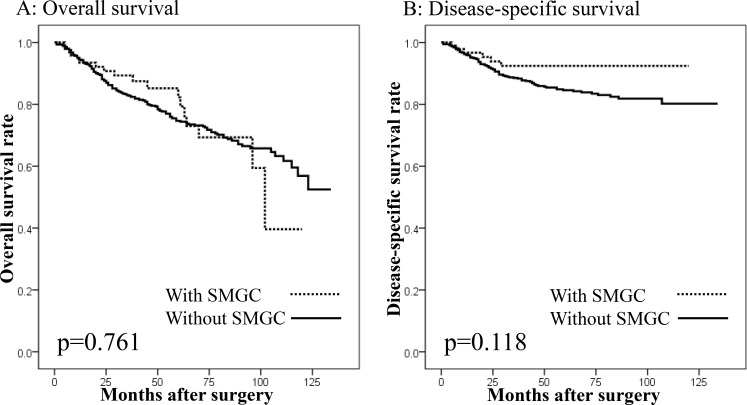
Survival curves of patients undergoing gastrectomy with curative intent according to the presence of synchronous multiple gastric cancers (SMGC) (**A**) Overall survival. No difference in overall survival was seen between patients with and without SMGC (*p* = 0.761). (**B**) Disease-specific survival. No difference in disease-specific survival was seen between patients with and without SMGC (*p* = 0.118).

**Figure 3:**
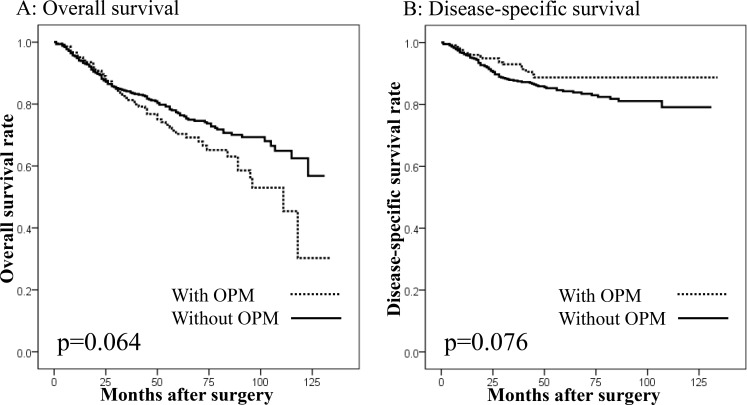
Survival curves in patients undergoing gastrectomy with curative intent according to the presence of other primary malignancies (OPMs) (**A**) Overall survival. No difference in overall survival was seen between patients with and without OPMs (*p* = 0.064). (**B**) Disease-specific survival. No difference in disease-specific survival was seen between patients with and without OPMs (*p* = 0.076).

**Figure 4:**
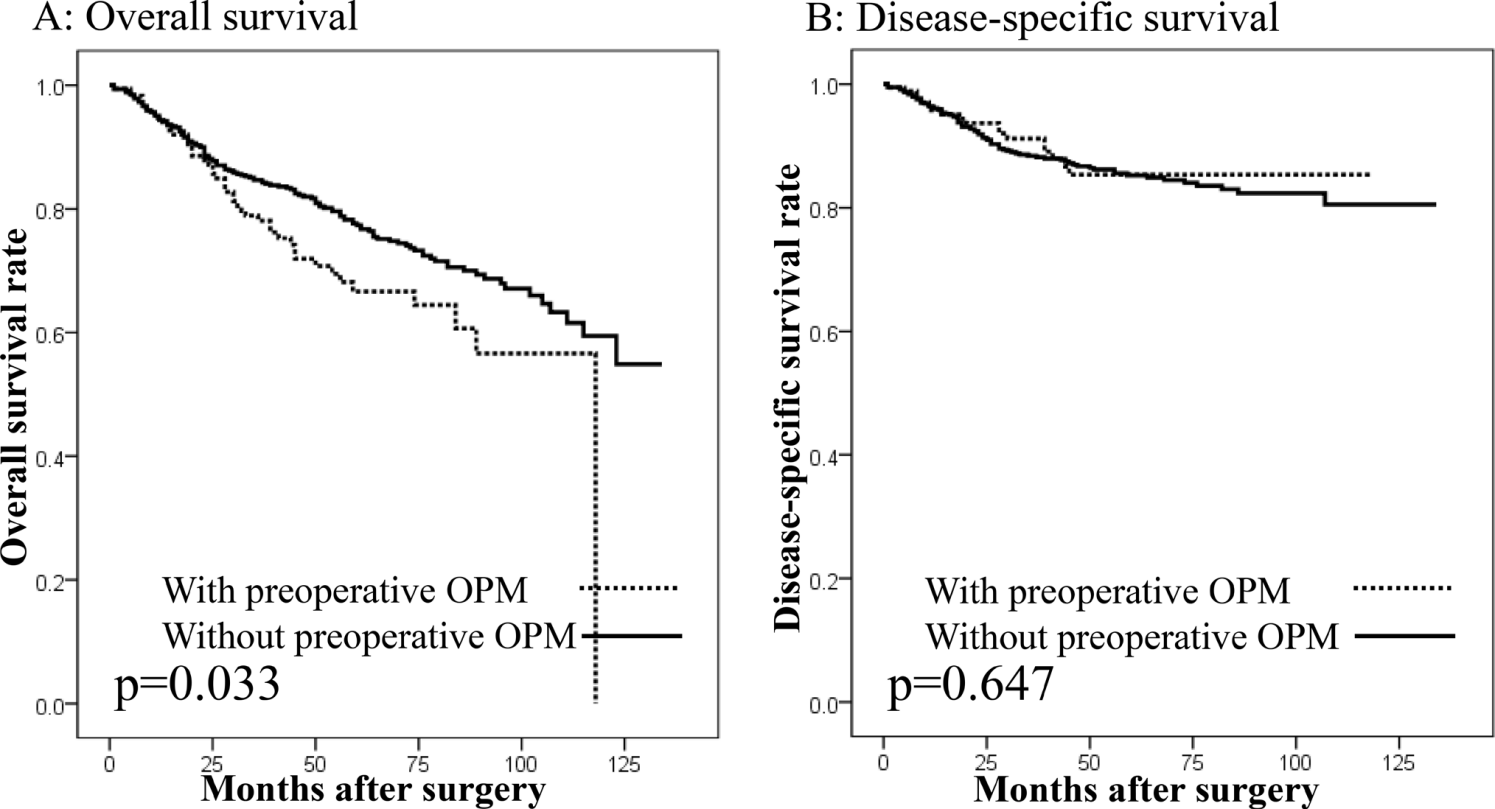
Survival curves in patients undergoing gastrectomy with curative intent according to the presence of other primary malignancies (OPMs) preoperatively (**A**) Overall survival. Patients with preoperative OPMs showed worse overall survival than those without (*p* = 0.033). (**B**) Disease-specific survival. No difference in disease-specific survival was seen between patients with and without preoperative OPMs (*p* = 0.647).

There were 995 patients undergoing gastrectomy with curative intent for GC. In these patients, there were no differences in the overall survival (OS) or disease-specific survival (DSS) between the patients with (*n* = 100) and without SMGC (*n* = 895: Figure 2A and 2B), or between patients with or without OPMs (Figure 3A and 3B). However, a significantly worse OS was observed in patients with preoperative OPMs than in those without (Figure 4A), whereas there was no difference in DSS (Figure 4B). In the multivariate analysis, although the presence of SMGC was not a significant predictive factor, preoperative OPM was identified as an independent prognostic factor in patients who underwent gastrectomy with curative intent (*p* = 0.021; HR: 1.481, 95% CI: 1.060–2.070; Table 9).

**Table 9:** Univariate and multivariate analysis on overall survival in gastrectomized patients with curative intent

Variable	Univariate analysis			Multivariate analysis		
	*p*-value	Hazard ratio	95% CI	*p*-value	Hazard ratio	95% CI
Age (year)	<0.001	1.05	1.034–1.066	<0.001	1.04	1.024–1.055
Sex (male)	0.053	1.367	0.996–1.878			
BMI (kg/m^2^)	0.001	0.93	0.890–0.972	0.104	0.964	0.923–1.008
SMGC (with)	0.761	0.928	0.571–1.506			
Preoperative OPM (with)	0.035	1.426	1.026–1.983	0.021	1.481	1.060–2.070
Histologic type (undifferentiated)	0.093	1.266	0.960–1.666			
Tumor size (mm)	<0.001	1.015	1.011–1.018	0.005	1.007	1.002–1.012
Tumor depth (pT2 or more)	<0.001	3.586	2.640–4.872	<0.001	1.964	1.368–2.820
Node metastasis (pN1 or more)	<0.001	3.529	2.662–4.680	<0.001	2.03	1.455–2.831

CI, confidence interval; BMI, body mass index; SMGC, synchronous multiple gastric cancer; OPM, other primary malignancy.

## DISCUSSION

Despite great efforts of gastrointestinal endoscopists to detect second or third lesions of GC, it is difficult to avoid missing lesions in cases of SMGC [7]. Various function-preserving procedures, such as endoscopic resection and limited gastrectomy, are performed for the treatment of GC [8]. However, these procedures lead to a larger remaining area of gastric mucosa with malignant potential in GC patients. Previous reports have demonstrated that SMGC are observed in 4–10% of GC patients [1, 7, 9], and the present study also showed a similar proportion. There are several risk factors for SMGC, such as advanced age, differentiated histology, and early GC [1]. Accordingly, in the present study, these factors were identified as independent risk factors of SMGC, consistent with previous studies.

Previous studies have reported an incidence of OPMs of 2.6–4.7% in GC patients [10–12]. Green *et al.* [13] reported that an OPM was observed in approximately 8% of advanced GC patients and 32% of early GC patients. Our previous study also showed that OPM was observed in 25% of GC patients [14]. In the present study, the incidence of OPM was 21.5%. The rate was especially high for antecedent and synchronous OPMs, which was probably because of the high proportion of elderly patients in our study.

In these situations, a relationship between OPMs and SMGC is currently being examined. Ojima *et al.* [5] reported that the presence of SMGC is a risk factor of synchronous colorectal cancer, while Kim *et al.* [6] reported SMGC as a predictive factor for future metachronous OPMs. Taken together, these studies suggest the presence of a common oncological or epidemiological factor that causes both MGC and OPMs. Miyoshi *et al.* [15] reported that microsatellite instability due to mutations of mismatch repair genes plays an important role in the development of MGC. Such genetic disorders have been demonstrated to be associated with the development of colorectal cancer, as well as other cancers, such as esophagus, thyroid, and prostate cancers, among others [16–20]. A genetic disorder, like a mismatch repair gene mutation, may be a common factor underlying the development of MGC and OPMs. The present study demonstrated that the presence of SMGC is a risk factor for the occurrence of OPMs postoperatively, as well as preoperatively. Several OPMs, including thyroid, esophagus, and colorectal cancers, were noted significantly more frequently in SMGC patients, both preoperatively and postoperatively, compared to in non-SMGC patients. Therefore, SMGC patients should be carefully assessed for the presence of these cancers, and gastrointestinal endoscopists, gastroenterologist, and surgeons should keep the relationship between SMGC and OPMs in mind.

The major determinant of prognosis in GC patients is the cancer stage, rather than the lesion number [1]. Our results showed that there were no differences in OS or DSS between the patients with or without SMGC. On the other hand, the presence of a preoperative OPM was an independent prognostic factor for OS. Our previous study also showed that GC patients with synchronous OPMs had a worse outcome after surgery than those without it [14]. Furthermore, Kim *et al.* [6] regarded that the presence of synchronous and metachronous OPMs negatively affected the clinical outcome of GC survivors. Our results suggest that OPMs may be associated with an increased risk of death. To improve the outcomes of GC patients, clinicians should make particular efforts to detect OPMs in patients with SMGC. It is important to consider the possibility of OPMs, especially in the organs where OPMs frequently occur. Routine esophagogastroduodenoscopy and colonoscopy are useful for detecting esophageal and colorectal cancers, respectively. However, the detection of cancers such as prostate and thyroid cancers is associated with a number of problems, including risks of overdiagnosis and overtreatment [21, 22]. Establishment of a standardized screening method for the detection of OPMs could contribute to improved survival in SMGC patients.

Our study has some limitations. First, our data included no information regarding the patients’ familial history, staging of OPMs, and *Helicobacter pylori* infection status. *Helicobacter pylori* cause chronic gastritis, leading to cancer in the stomach [23], and might contribute to the incidence of MGC. Second, the follow-up period was not enough to detect all future GCs or OPMs.

In conclusion, because patients with preoperative OPMs have a high risk of SMGC, careful preoperative examination is recommended to improve the patients’ prognosis.

## MATERIALS AND METHODS

### SMGC definition and screening

SMGC was assessed by pathologic examination using the resected stomach samples, and the results were compared with the preoperative esophagogastroduodenoscopy findings. Multiplicity of GC was determined by the criteria of Moertel *et al.* [24], as follows: (1) two or more GCs must be pathologically proven to be malignant, (2) all lesions must be separated macroscopically by an area of normal gastric wall, and (3) the possibility that one of the lesions represents local extension of a metastatic tumor must be ruled out beyond reasonable doubt. Synchronicity of MGC was defined according to the criteria of Warren and Gates [25]. When two primary cancers were detected within 1 year, they were considered synchronous. When two primary cancers were detected more than 1 year apart, they were considered metachronous. The pathological features of the MGC were defined by main lesion, which is more advanced lesion and to be larger lesion if the depth of invasion was same.

### OPM definition and screening

Multiple primary cancers arising from other organs were also defined according to the criteria of Warren and Gates [25]. Metachronous multiple primary malignancies included antecedent malignancies before surgery for GC and subsequent malignancies after surgery for GC. In order to identify preoperative risk factors for MGC, we classified the OPMs into two groups: a preoperative group including antecedent and synchronous OPMs, and a postoperative group including subsequent OPMs.

The histopathologic findings of GC were obtained using the resected specimens. The clinicopathologic features of GC were described according to the TNM classification (7th edition).

Before surgery for GC, we performed total colonoscopy when patients were able to consume food. When colonoscopy could not be performed preoperatively, we performed it within 1 year after surgery. Furthermore, we performed whole-body computed tomography in order to detect OPMs. When malignancies were suspected in other organs, we added several examinations specific to the suspicious organs. After gastrectomy, the GC patients were followed in the outpatient clinic of each hospital not only to check for recurrence and metastasis of GC, but also to detect OPMs, by esophagogastroduodenoscopy, colonoscopy, and whole-body computed tomography. Furthermore, when the patients complained of symptoms other than those in the abdomen, we consulted clinical specialists.

### Statistical analysis

Data are shown as the prevalence (%) or mean values. Continuous variables were compared using the Mann-Whitney test, and categorical variables were compared using the Chi-square test or Fisher’s exact test, depending on their distribution. Multivariate analysis of independent risk factors was carried out by multiple logistic regression tests, and prognostic factors were analyzed by Cox’s proportional hazard models. The survival rates after gastrectomy were calculated by the Kaplan-Meier method. The data were analyzed using IBM SPSS 22.0 (IBM Japan, Tokyo, Japan). For all analyses, *P* < 0.05 was considered significant. Missing data were accounted for using a list-wise deletion approach.

## Abbreviations

CI: confidence interval
DSS: disease-specific survival
GC: gastric cancer
HR: hazard ratio
MGC: multiple gastric cancers
OPM: other primary malignancy
OS: overall survival
SMGC: synchronous multiple gastric cancers
